# The State of Cancer Care in the United Arab Emirates in 2022

**DOI:** 10.3390/clinpract12060101

**Published:** 2022-11-23

**Authors:** Humaid O. Al-Shamsi

**Affiliations:** 1 Department of Oncology, Burjeel Cancer Institute, Burjeel Medical City, Abu Dhabi P.O. Box 92510, United Arab Emirates; alshamsi@burjeel.com or humaid.al-shamsi@medportal.ca; Tel.: +971-50-631-5388; 2 Innovation and Research Center, Burjeel Cancer Institute, Burjeel Medical City, Abu Dhabi P.O. Box 92510, United Arab Emirates; 3 College of Medicine, University of Sharjah, Sharjah P.O. Box 27272, United Arab Emirates; 4 Emirates Oncology Society, Dubai P.O. Box 6600, United Arab Emirates

**Keywords:** United Arab Emirates, incidence, cancer, screening, onco-fertility, psycho-fertility, survivorship, medical tourism, COVID-19, palliative care

## Abstract

Cancer is the third-leading cause of death in the United Arab Emirates (UAE); cancer care in the UAE has evolved dramatically over the last 40 years, from a single center in Al Ain in 1981 to more than 30 cancer centers and clinics across the UAE, with at least four comprehensive cancer centers in the UAE nowadays. Despite the significant progress in medical care, cancer quality control across the UAE is still lacking, with significant variations in cancer care across the cancer centers. Access to clinical trials is still hampered by a lack of expertise and research infrastructure and a small population, which renders patient accrual for trials a major challenge. Education and training are other areas for improvement that require immediate attention, and, in this review, we try to address these critical aspects for stakeholders to consider better cancer care in the UAE. Early cancer detection and screening are still evolving in the UAE, and a national screening program is lacking. There is also a need to address barriers to screening and to consider less invasive screening methods such as approved blood-based screening, which is likely to be more acceptable to the UAE population. In this review, we also address new topics that have not been addressed previously, including oncology medical tourism, psycho-oncology, onco-fertility, precision oncology, survivorship, oncology nursing, cancer support programs, and the oncology sector’s response to the COVID-19 pandemic, all in the context of the UAE cancer landscape. Finally, we provide recommendations for policymakers, regulators, payers, patient advocacy groups, and the UAE oncology community regarding the delivery and future planning of high-quality cancer care. These recommendations are aligned with the UAE government’s vision to reduce cancer mortality and provide high-quality healthcare for its citizens and residents.

## 1. Introduction

Cancer is one of the major health issues in the United Arab Emirates (UAE), leading to substantial morbidity and mortality. It accounts for 10% of all mortality in the UAE, which made it the third-leading cause of death after cardiovascular disease and injuries in 2019 [1]. The UAE is determined to reduce cancer mortality by nearly 18% by 2025. Reducing the number of deaths due to cancer is one of the key performance indicators of the UAE’s national agenda as a “Pillar of World-Class Healthcare” [2].

There are limited data available regarding the current status of cancer care in the UAE. These data are critical to identifying gaps and to improving cancer care in the UAE [2,3,4]. This review is the most comprehensive review of cancer care to date, covering many previously unexplored topics, such as oncology medical tourism, psycho-oncology, onco-fertility, precision oncology, survivorship, oncology nursing, cancer support programs, cancer education and training, cancer research in the UAE, and many others. This review provides recommendations for all stakeholders, including policymakers, regulators, payers, and the UAE oncology community, regarding the delivery and future planning of high-quality cancer care.

## 2. History of Cancer Care in the UAE

**Figure 1:**
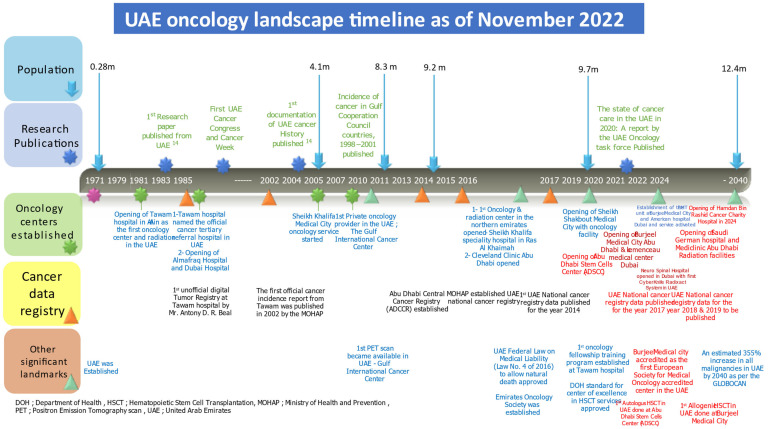
UAE oncology landscape timeline as of November 2022.

We have previously outlined the historical background of cancer care in the UAE. The history of cancer care in the UAE started with the opening of Tawam hospital in Al Ain in 1981 [2]. The most significant advances in the UAE in the last few years include the introduction of cyberknife radiation and the initiation of hematopoietic stem cell transplantation (HSCT) [5,6]. Furthermore, additional cancer centers have opened. Figure 1 presents an outline of the UAE oncology landscape.

## 3. Incidence of Cancer in the UAE in 2019

Between 1 January and 31 December 2019, the total number of newly diagnosed cancer cases (malignant and in situ) reported to the UAE National Cancer Registry (UAE-NCR) was 4633 [1]. Of these, 4381 (94.56%) were malignant, and 252 (5.44%) were in situ cases (Appendix A). Cancer was more common among women than men; it affected 2604 (56.2%) females and 2029 (43.8%) males. Among UAE citizens, 1193 cases were newly diagnosed with cancer, out of which 1117 (93.6%) were malignant and 76 (6.4%) were in situ cases. Similarly, in non-UAE citizens, 3440 cases were newly diagnosed cancer, 3264 (94.9%) were malignant, and 176 (5.1%) were in situ cases, representing an overall crude incidence rate of 46.1/100,000 for both genders. The figures showed a clear female predominance in cancer incidence. The crude incidence rate was higher for females, at 75.8/100,000, than for males, at 31.0/100,000. The overall age-standardized incidence rate (ASR) was 78.4 per 100,000. Breast, thyroid, colorectal, skin, and leukemia were the top-ranked cancers among all new cancer cases in both genders (Table 1). Colorectal, skin, prostate, leukemia, and non-Hodgkin’s lymphoma were the top-ranked cancers among males (Table 2). Among females, breast, thyroid, colorectal, uterus, and ovary cancers were the top-ranked cancers (Table 2). In 2019, 125 children in the age group of 0–14 years were diagnosed with new cancer in the UAE (54% were females and 46% were males). This constituted about 2.9% of all registered malignant cases. Leukemia, brain and CNS, connective and soft tissue, non-Hodgkin’s lymphoma, and bone and articular cancers were the most common cancers in boys and girls. The third-leading cause of death in the UAE, after diseases of the circulatory system and injuries, was found to be cancer. The number of deaths from cancer totaled 1181 (629 in males, 552 in females) and accounted for 13.11% of all deaths, regardless of nationality, type of cancer, or gender (Table 3). This represents an estimated age-standardized mortality rate of 33.3 deaths per 100,000 for both genders. Breast cancer was the leading cause of cancer death in 2019, with an estimated average of 11.6% of cancer deaths yearly. Colon cancer was the 2nd most common cause of cancer death in both sexes. Lung cancer was the third most common cause of cancer death in both sexes (Table 3).

## 4. Cancer Care for Young Adults and Adolescents

There is accumulating evidence that the incidence of cancer is increasing in younger adults [7]. In our previous report, the young population in the UAE indicated a higher incidence rate of breast and colorectal cancer [8,9]. An open-access dataset for 2017 was extracted and released from the UAE National Cancer Registry (UAE-NCR) at the Ministry of Health and Prevention. We extracted and investigated data for the year 2020 from SEER* statistics for the USA and from the International Agency for Research on Cancer (IARC) for Canada, the United Kingdom, Saudi Arabia, China, and India. The analysis of the UAE-NCR’s published data for 2017, with a concentration on the age group between 20–49 years for both UAE and non-UAE citizens, indicated that the percentage of cancer incidence among this age group in 2017 was 45.4% of the total number of new cancer cases, compared to 42.7% and 42.4% in 2016 and 2015, respectively. The percentage of cancer incidence among females from this age group in 2017 was 51.3% of the total number of new cancer cases and 38.3% in males for the same year, compared to data extracted from the years 2016 and 2015, which indicated 49.5% females and 34.9% in males and 49.9% females and 34.1% in males, respectively. A coherent trendline showing an increase of 3% in the annual incidence from 2015 to 2017, from 42.4% to 45.4%, respectively, in this age group, was observed. This increased incidence rate persisted irrespective of sex, with a 4.2% increased incidence in males observed from 2015 to 2017—i.e., 34.1% to 38.3%, respectively—and a 1.4% increased incidence in females observed from 2015 to 2017, i.e., 49.9% to 51.3%, respectively. This means more females between the age of 20 to 49 compared to their male counterparts were diagnosed with cancer between 2015 and 2017.

Upon re-analyzing the data from 2017 and restricting the search based on nationality (UAE citizen) and age group (20–49), the cancer incidence percentage remained high at 37.2% (42.4% in females and 28.9% in males). A comparable percentage of cancer incidence was observed for the 20–49 year age group in Saudi Arabia, which was 39.49%. These incidence rates were massively high when compared to other countries, such as the USA at 8.75% (*p* < 0.005), Canada at 8% (*p* < 0.005), the United Kingdom at 8.33% (*p* < 0.005), India at 26.75% (*p* < 0.005), and China at 16.15% (*p* < 0.005).

According to the current data, cancer incidence has increased in the UAE among people aged between 20 and 49. This alarming incidence rate demands more intensive research to identify potential risk factors. Cancer screening is the most vital component that reduces cancer mortality, yet its cost-effectiveness and utility have not been thoroughly assessed among the younger population (<40 years). UAE-based research is necessary in order to assess the country’s monitoring and regulatory system for cancer screening. More cohesive regional and international efforts are required to combat this globally alarming situation.

## 5. Cancer Screening Programs in the UAE

The UAE has made significant advances in cancer screening programs, yet a UAE-wide national screening program is still lacking. There are several local screening programs for each region, as outlined in Table 4 [10,11,12,13,14]. Unfortunately, no official published data are available on cancer screening uptake, but unofficial reports estimate that breast and colorectal cancer screening is used by no more than 25% of the eligible population [15]. Instead of the current opportunistic screening approach, there is a need for a UAE-wide national screening program that covers breast, colorectal, cervical, lung, and prostate cancer and includes a call-and-recall system [13,16,17]. Screening for citizens vs. non-citizens should also be evaluated as 73% of all cancer cases occur in non-citizens vs. 27% in UAE citizens [18]. Hence, currently, most of the cancer care expenditure is for non-citizens. Social barriers to cancer screening must be addressed systematically [15,19,20,21]. The utilization of approved blood-based cancer screening (e.g., Epi proColon) can be considered as an option that may be less invasive and has potential higher uptake for cancer screening [22]. Consideration for participation in active clinical trials for blood-based cancer screening tools should be prioritized [23,24,25].

## 6. Modifiable Cancer Risk Factors in the UAE (Obesity/Fast Food/Smoking/HPV/HBV)

The prevalence of obesity and overweight in UAE youth has been found to be consistent with other Arab countries in the Middle East, owing to the increased consumption of unhealthy diets with poor nutritional benefits [26]. We have previously addressed the UAE’s efforts to control risk factors for cancer. Obesity and smoking (including all forms) have been on the UAE government’s active agenda, with multiple initiatives to control them [2]. We also recommend displaying calories in all fast-food items and banning advertising for fast food restaurants, as this is becoming very prominent online and in the UAE’s streets [27].

Cervical cancer is the 8th most common female cancer in Abu Dhabi [28]. The UAE was the first in the MENA region to launch the HPV vaccine. In 2008, the Department of Health (DOH) in Abu Dhabi introduced HPV vaccination for all eligible schoolgirls in public and private schools. In 2013, the DOH expanded the HPV vaccination program to include all females aged 15–26, regardless of nationality. The Dubai Health Authority (DHA) also recommended starting the vaccination of all girls at the age of 11–12 years [2]. There are no official reports available regarding the uptake of the vaccination since its introduction. Multiple reports and cross-sectional studies from the UAE indicate the likelihood of a low uptake due to a lack of knowledge of HPV vaccination [2,29,30,31]. We recommend improving the public, parental, and adolescent education about HPV vaccination, specifically to reduce the misconceptions and fear surrounding HPV vaccination [2,32].

The Hepatitis B vaccine was introduced and mandated in 1991 in the UAE [33]. Since 2006, any new expats moving to the UAE for work must be tested for hepatitis C and B infections. If the subject is not HBV-immune, it is mandatory to receive an HBV vaccination [2].

## 7. Established Comprehensive Cancer Centers

There are over 20 oncology centers/clinics across the UAE. We have previously outlined the historical background of major cancer centers in the UAE [2]. There is no official definition of a comprehensive cancer center (CCC) in the UAE. The DOH has published general standards for centers of excellence in the emirate of Abu Dhabi that are not specific to oncology [34].

A CCC should be a one-stop shop for all cancer care needs. [35] In our view, the following services must be available at a facility for it to be considered a CCC: medical adult and pediatric oncology and hematology, surgical oncology, radiation oncology, nuclear medicine, and palliative care [35]. Currently, four centers can be considered CCCs (Table 5). Other major cancer centers that do not meet our CCC criteria are Dubai Hospital, Saudi German Hospital Dubai, Sheikh Shakhbout Medical City, Cleveland Clinic Abu Dhabi, and Mediclinic Hospital Abu Dhabi. Cancer centers and hospitals in the UAE are listed in alphabetical order in Appendix B.

The Al Jalila Foundation, which is a member of Sheikh Mohammed Bin Rashid Al Maktoum Global Initiatives, is planning to establish the first comprehensive cancer charity hospital in Dubai, ‘Hamdan Bin Rashid Cancer Charity Hospital’, bringing together leading experts to manage prevention, diagnosis, and treatment under one roof. The hospital, named after the late Sheikh Hamdan Bin Rashid Al Maktoum, will provide outpatient, ambulatory, and diagnostic services, as well as inpatient and surgical services in a nurturing environment that prides itself on personalized patient care. Patients will be accepted from anywhere in the UAE, and the medical services provided will either be free or highly subsidized to alleviate the financial burden for patients who cannot afford quality healthcare. The Al Jalila Foundation is investing AED 750 million in the first fully modular-built hospital in the region. The 250-bed hospital will be built in two phases, with phase 1 including 150 beds. Opening in 2024, the facility will be a one-stop-shop for cancer care: prevention, diagnosis, and treatment, with a capacity to treat 30,000 patients annually [36].

## 8. Oncology Manpower in the UAE

There are an estimated 100 medical oncologists, radiation oncologists, and malignant hematology physicians in the UAE as of August 2022. Most oncologists are in the Abu Dhabi and Dubai emirates. Oncologists come from various nationalities, with many from the UK, the USA, and Arab countries such as Lebanon, Syria, and Jordan. Many have relocated to the UAE for better pay and the quality of life that the UAE provides. Oncologists come from different training backgrounds, with many holding American and UK board certifications. There are also many oncologists with training from neighboring countries such as Lebanon, Jordan, and Syria. The number of local UAE oncologists is around 10–12 (10% of all oncologists in the UAE). Most local UAE oncologists are Canadian- and American-trained physicians, including advanced fellowships from top institutions such as MD Anderson Cancer Centers. There is a need to encourage UAE physicians to pursue careers in oncology by increasing awareness about the specialty among medical students and physicians early in their careers.

## 9. Surgical Oncology and Robotic Surgeries

Surgical oncology has been practiced in the UAE for decades, yet many surgeons practicing surgical oncology in the UAE have not received formal training in surgical oncology. More formally trained surgical oncology surgeons have been joining the UAE steadily over the last few years. It is well documented that the surgeon’s training affects the outcomes of cancer patients. In 27 studies examining surgeon outcomes based on training and specialization, 25 found that specialized surgeons had better outcomes for cancer surgery than non-specialized surgeons [37]. The UAE has increased access to robotic surgery, with few cancer centers providing this modality.

One of the significant challenges of surgical oncology in the UAE is the quality of surgeries performed for cancer patients. Unfortunately, many surgeons with no surgical oncology training will not refer their patients to specialized surgical oncologists for surgeries due to financial conflicts of interest and competition (with most working in private practices). Surgeries not carried out by surgeons who specialize in surgical oncology are usually not discussed with a multidisciplinary team (MDT). This leads to improper surgical management, especially in breast and rectal cancers, where patients are operated on while they need neoadjuvant therapy to improve their outcomes.

The regulators need to address this issue by mandating that an MDT must evaluate all cancer cases before any insurance approval is provided for cancer surgeries (unless it is an emergency surgery, and surgeons who carry out a high number of emergency cancer surgeries must be audited). Furthermore, the use of robotic surgery should be restricted to surgeons with accredited surgical training [38] as some surgeons attend courses for a few days and advertise themselves as robotic surgeons, which can affect cancer patients’ decisions to receive surgery.

## 10. Radiation Therapy in the UAE

Currently, at least ten centers are providing radiation therapy services in the UAE; four in Abu Dhabi and Al Ain, five in Dubai, and one in Ras Al-Khaimah (the only radiation center in the Northern Emirates). These centers are currently operating seven linear accelerators (LINACs), one tomotherapy unit, and two brachytherapy units in the Abu Dhabi/Al Ain region. Currently, there are two linear accelerators and one ViewRay MR Linac in Ras Al-Khaimah, whereas in Dubai, there are four linear accelerators, one tomotherapy unit, one Cyberknife machine, and two brachytherapy units in use. Most LINACS in the UAE are of recent generation and new status, with Elekta Versa HD™ and Varian—TrueBeam^®^ being the most widely used in the UAE. Complex intensity-modulated radiation therapy (IMRT), RAPID-ARC, and volumetric modulated arc therapy (VMAT), accompanied by advanced image guidance radiation (IGRT), are used in all of these sites. Cyberknife radiosurgery was introduced into Neurospinal hospital in Dubai in 2022 [5]. A full Novalis system with BrainLab and Elements software, a tool for delivering precision radiotherapy and stereotactic radiosurgery (SRS), was introduced into the UAE by Burjeel Medical City in 2022 [39]. Finally, Ras Al-Khaimah is home to the Middle East’s only ViewRay—MRIdian, the first FDA-cleared MRI-Guided Radiation Therapy cancer treatment system in the Middle East, which combines magnetic resonance imaging with adaptive radiotherapy [40].

The field of radiation therapy in the UAE is growing and has been successful in terms of its quality and outcomes. In the short term, at least four more centers are planning to establish radiation facilities in the UAE with advanced technologies such as gamma knife and artificial adaptive planning LINACs. Moreover, proton therapy establishment has been planned at one center, yet previous attempts to bring proton therapy to the UAE have been hampered by high cost and a low number of eligible patients for proton therapy [41]. While this indicates successful radiotherapy provider expansion and growth, we believe that further restrictions on more radiation centers will be recommended to ensure that current practices maintain the higher quality associated with a high volume of treatment.

Another critical aspect of improving radiation therapy in the UAE is establishing an independent, non-biased regulator quality-control program for radiation facilities across the UAE. Adopting a change of the payment model to “per-site” rather than “per-fraction” is also highly recommended to eliminate any potential unnecessary negative clinical and financial effects on patients and healthcare systems while maintaining high quality and enhancing efficiency. The potential for fewer treatments under the episode-based payment approach will likely lead to reduced travel time required for each treatment, reduced side-effects from treatment, less time spent in a physician’s office, and more free time to engage in other social activities that can help improve patients’ overall quality of life [42].

## 11. Hematopoietic Stem Cell Transplantation

Hematopoietic stem cell transplantation (HSCT) has become a well-established, curative, or lifesaving treatment option for various non-malignant and malignant hematologic diseases, immune disorders, and solid tumors. A total of 164 pediatric and 161 adult citizen patients underwent HSCT outside the UAE between 2016 and 2018 [6]. An estimated 200 patients, including non-citizens, need HSCT annually in the UAE.

The first HSCT service has been available in the UAE since 2019, when the Abu Dhabi Stem Cell Center established it, and as of August 2022, they have completed 11 low-risk autologous non-cryopreservation HSCTs. In October 2021, Burjeel Medical City established the most comprehensive HSCT unit for both adults and pediatrics and introduced cryopreservation HSCT to the UAE with 14 cryopreserved autologous cases, as well as completing the first pediatric allogeneic HSCT with five cases. The American Hospital Dubai (AHD) became the third provider to deliver HSCT in the UAE in December 2021 and has completed nine autologous HSCT cases. Of note, all the above providers are private healthcare providers. Sheikh Shakhbout Medical City, a public hospital, has been planning an HSCT service for over a year, with an expected launch of their service late in 2022 or early 2023.

With a small estimated potential for HSCT cases annually in the UAE, it is critical to establish a national HSCT center of excellence and limit the number of providers to allow the experience to accumulate in one reference center for the UAE. We also recommend establishing a UAE National Marrow Donor Program to facilitate lifesaving HSCT for patients who do not have a compatible donor in their family.

Currently, CAR-T cell therapy is not offered in the UAE. Burjeel Medical City will be the first center to deliver this modality in the next 24 months to expand its BMT and cellular therapy programs.

## 12. Gynecologic Oncology

Women’s cancers were the 4th most common cancers in the UAE in 2019, with 125 cases of uterine cancer, 100 cases of ovarian cancer, and 90 cases of cervical cancers, for a total of 315 cases of women’s cancers (7.1% of malignancies in the UAE in 2019). General gynecologists and general surgeons operate on many women’s cancer cases. Currently, there are no dedicated gynecologic oncology units in the UAE. Moreover, the number of trained gynecologic oncology surgeons is very limited and is estimated to be less than ten across the UAE. There is a lack of regulations regarding the limitation of the general surgeon’s management of gynecologic oncology cases, which has been shown to lead to suboptimal clinical and oncological outcomes [43]. We recommend establishing dedicated gynecologic oncology units, mandating referrals to these units across the UAE, and restricting general surgeons from performing gynecologic oncology surgeries.

The low uptake of cervical cancer screening should also be addressed, and research should address barriers to screening. The HPV vaccine, as discussed earlier, is part of the UAE’s national vaccination program for girls, with a successful implementation. Lastly, there are no gynecologic oncology fellowship training programs in the UAE, and we highly recommend establishing such programs.

## 13. Pediatric Oncology

Cancer has been identified as the second most prominent cause of death (following accidents) in children aged 5 to 14 [44]. The incidence of pediatric cancers differs worldwide, representing between 0.5% and 4.6% of all cancers. Overall incidence rates fluctuate between 50 and 200 per million children worldwide [45]. Childhood cancer results in a substantial disease burden despite a relatively low absolute number of incident cases and deaths.

According to the UAE-NCR [1], in the year 2019, there were 125 children in the age group of 0–14 years diagnosed with new cancer in the UAE. This accounts for approximately 2.9% of all registered malignant cases, with females accounting for 53.6% and males accounting for 46.4%. The data indicate that the highest frequency of pediatric cancer cases was found among the age group of 0–4 years (63; 50.4%), followed by the age group of 10–14 years (38; 30.4%). It was noted that a smaller number of cancer cases in the pediatric population was diagnosed in the age group of 5–9 years (24; 19.2%).

The data indicate that the most commonly occurring cancer was leukemia (35.2%), followed by brain and CNS (11.2%), connective and soft tissue (7.2%), and non-Hodgkin’s lymphoma (7.2%), and bone and articular cartilage cancer (5.6%) [1] (Table 6).

Seven hospitals provide pediatric hematology oncology services, with an estimated 28–30 hematology oncology physicians. Tawam hospital, Sheikh Khalifa Medical City, and Dubai hospital are the public hospitals providing these services and these have the highest patient flow. Of note, the Northern Emirates has no pediatric hematology oncology services (Table 7).

Burjeel Medical City is the only hospital in the UAE with a BMT pediatric unit and this facility performed five successful allogeneic BMTs for the first time in the UAE, with the first procedure performed in April 2022 [46].

Pediatric hematology oncology services are fragmented between many providers for a small patient population. We recommend centralizing pediatric hematology oncology services in the UAE for better treatment outcomes [47].

## 14. Palliative and Supportive Care

In the four years since the publication of the previous report titled “Palliative care in the UAE, A desperate need” in 2018 [48], palliative and supportive care have come a long way in the UAE, growing from two centers providing palliative care to four centers across the UAE. The palliative care unit at Tawam hospital was opened in 2007 to serve oncology patients at Tawam hospital, and this remains the only government-funded palliative care program in the UAE. The American Hospital in Dubai commenced its palliative care service in December 2014 [48]. Mediclinic hospital Dubai started the service in 2019. Burjeel Medical City (BMC) in Abu Dhabi was the latest hospital to offer a palliative and supportive care service in March 2020, becoming the first provider of such a service in Abu Dhabi City. In May 2022, the UAE received its first representative in the WHO’s palliative care network; Dr. Neil A. Nijhawan, the director of the palliative care services at Burjeel Medical City in Abu Dhabi, was appointed by the WHO as an expert member of the EMRO Expert Network on Palliative Care.

The Emirates Medical Association (EMA) has approved the establishment of a palliative and supportive care working group under the Emirates Oncology Society (EOS) umbrella. The working group is planned to be launched in October 2022. This is an important step to raise public awareness about palliative care and advocate for the provision of palliative care in the UAE. The working group will also play an integral part in advocacy for the specialty with the stakeholders and regulators in the UAE. Education and training will also be a core mission for the group.

Improving the provision of palliative care within the UAE will require a more nuanced, structured approach than a simple transfer of the Western approach to palliative care. Our recommendations for the UAE regarding the improvement of palliative care were highlighted in our previous publication. These have been refined further as follows [49]:

- The development of a national palliative and supportive care strategy as part of the UAE’s cancer control plan;
- The availability of essential pain and palliative care medications at all levels of care, including injectable opioids/morphine pumps for patients receiving end-of-life care at home;
- Basic palliative care training for non-specialists, with a focus on pain management, as well as palliative care in undergraduate medical school and nursing curricula;
- Provisions for other core multidisciplinary team member roles that require training and support (governance) include clinical nurse specialists, imams, and chaplaincy; and
- Further refinement of the 2016 Allow Natural Death (AND) policy to include advance care planning and treatment-de-escalation plans [49].

## 15. Cancer Survivorship Program

Cancer survivorship is an essential yet poorly recognized component of cancer care [50]. It revolves around short- and long-term treatment complications; the risk of recurrence; the potential increased risk of second primary malignancies; failure of adherence to prescribed adjuvant therapies; and recommended lifestyle changes, including weight loss and control, physical activity, and exercise [50,51]. Cancer survivorship programs remain in their infancy in the UAE, with only two centers having comprehensive cancer survivorship programs, namely, Burjeel Medical City and Tawam Hospital, with a clear unmet need in other emirates. Existing programs also need to publish experiences and challenges to inform best practices. Stakeholders need to be made aware of the importance of survivorship programs to the continuum of care for cancer patients [52].

## 16. Onco-Fertility

With the global phenomenon of the increasing number of younger adults with cancer [53] and as the number of cancer patients in the UAE at the productive age is also increasing (as outlined earlier), fertility preservation is becoming increasingly important for these younger patients [54].

A recent study demonstrated a significant gap between international clinical practice guidelines and current practices in fertility preservation in Arab countries. Barriers to the optimum delivery of service include the unavailability of some advanced techniques, the lack of physician training/awareness, and the lack of a dedicated fertility team/clinic within cancer centers [55].

The main centers with the most experience in onco-fertility in the UAE are Al Ain Fertility Center and Fakih IVF Fertility Center. They provide cryopreservation (freezing embryos or eggs) and ovarian tissue freezing for female patients. The options for younger men with cancer include freezing sperm and/or testicular tissue. Other procedures for ovarian transposition, fertility-sparing surgery, and hormonal ovarian suppression are widely available in the cancer centers in the UAE.

Some major challenges are physicians’ awareness of the available resources for young adults with cancer for fertility preservation and insurance coverage, as many insurance policies exclude fertility preservation from their policies. This is the main issue for expat patients, whereas UAE citizens are covered for onco-fertility services.

Pre-implantation genetic diagnosis, which is the testing of pre-implantation stage embryos or oocytes for genetic defects, e.g., BRCA carriers, is not available in the UAE, and patients who require this modality are usually referred to specialized fertility centers abroad.

Focused workshops for oncology healthcare providers and participation by stakeholders, including providers, regulators, and patient advocacy groups, are required to improve fertility access for all cancer patients in the UAE.

## 17. Psycho-Oncology

With a new or recurrent cancer diagnosis, patients with cancer are at risk of developing emotional difficulties that range from adjustment disorder, loss of self-esteem, anxiety, and depression. It is estimated that up to 50% of cancer patients experience some form of emotional difficulty. If severe enough, emotional difficulties and distress can interfere with the patient’s ability to deal effectively with cancer, its symptoms, and treatment complications. The patients at higher risk for psychiatric illness and depression tend to have advanced cancer, poorly controlled pain, a previous psychiatric history, and other life losses or stressors [56]. Psycho-oncology concerns cancer’s social, behavioral, psychological, and ethical aspects. This subspecialty addresses the two major psychological dimensions of cancer: the psychological responses of patients to cancer at all stages of the disease and those of their families and caretakers, as well as the psychological, behavioral, and social factors that may influence the disease process [57].

In the UAE, psycho-oncology is still in its infancy. There are no specialists licensed or trained explicitly in psycho-oncology, yet there are many psychiatrists and psychologists providing support for cancer patients. There is a need for the establishment of psycho-oncology clinics across the cancer centers in the UAE to support cancer patients across the UAE more effectively. Attracting trained physicians, providing scholarships for psychiatry trainees to pursue psycho-oncology fellowships and advanced training, and working with regulators and healthcare providers to address this gap is the most appropriate next step of action to accelerate the implementation of psycho-oncology across the UAE.

## 18. Cancer Support Programs and Support Groups

Cancer support programs support cancer patients financially, whereas support groups provide psychological support. The primary issue with most financial support programs is that they have a monetary limit for each cancer patient, and in most cases, the amount covered is far less than what is required to cover the treatment cost. Indeed, there are a few exceptions with higher coverage for cancer patients. Most notably, the weekly TV show from Sharjah TV, “Alam W Amal”, which translates to “Pain and Hope,” can provide coverage of up to AED 180,000 per case. Of note, many pharmaceutical companies’ patient-support programs cover cancer drugs manufactured by those companies. This also plays an essential role in supporting cancer patients in need across the UAE. Multiple support programs help support the emotional and physical well-being of patients with cancer. Table 8 summarizes the cancer support programs and support groups available for cancer patients in the UAE.

## 19. Genetic Testing and Counseling

There is limited access to genetic counselors in the UAE. Most cancer centers do not have a dedicated genetic counseling section. Most counseling testing is performed by the oncologists providing treatment, with evident variations in skills and knowledge in this niche area of expertise. Most health insurances policies exclude genetic testing from their policies. Access to genetic testing is challenging for most cancer patients in the UAE. The few centers providing genetic counseling and testing have extended appointment wait times. Many tests are not covered, and if covered, testing is performed outside of the country and takes 4–6 weeks to provide results. To overcome the barriers to access to genetic testing, multiple pharmaceutical companies provide free genetic testing for some indications, such as BRCA testing for breast cancer patients. We recommend expanding genetic counseling services in cancer centers across the UAE with experienced genetic counselors. Regulators and stakeholders should work to make genetic testing widely available and accessible for all cancer patients, in keeping with international guidelines and recommendations for best practices in cancer care [58,59].

## 20. Precision Oncology in the UAE

Precision oncology, defined as the molecular profiling of tumors to identify targetable alterations, is rapidly developing and has entered mainstream clinical practice [60]. Actionable biomarkers of non-small-cell lung cancer are an excellent example of precision oncology at diagnosis of advanced disease, during therapy, and at the time of progression. Without precision oncology and mutation assessments, lung cancer treatment is suboptimal nowadays [61].

Precision oncology tools such as next-generation sequencing (NGS) testing are widely available and used by many oncologists in the UAE. Multiple commercial testing platforms are available, including Illumina Inc foundation medicine, the Guardant360^®^ system, and many other NGS platforms, but all of these tests are sent abroad for testing, and none are performed locally in the UAE. Almost all payers in the UAE do not cover these tests, except for a small percentage of premium private insurance providers, which may not exceed 5% to 10% of all cancer patients requiring NGS testing with the premium private insurance that covers these tests. The current commercially available NGS testing cost is also a significant barrier preventing patients from obtaining more accurate molecular profiling of their tumors. It is necessary for a cheaper and more reliable testing platform to be made available in the UAE. Multiple small multinational companies are providing more affordable NGS testing in the UAE, but more data are needed for these platforms before they can be considered a reliable alternative.

The Oncotype DX Breast Recurrence Score^®^ Test is a test that analyzes the activity of 21 genes that can influence how likely a cancer is to grow and respond to chemotherapy and predicts the risk of breast cancer recurrence in some early breast cancer cases. The test is commercially available in the UAE, but most payers do not cover it as it is considered genetic testing (which it is not) and is hence excluded from health insurance policies. The MammaPrint^®^ test analyzes the 70 most important genes associated with breast cancer recurrence and has recently been covered by some insurance companies. These tests should be covered by insurance in all eligible breast cancer cases in keeping with best practices and international recommendations [62].

One of the other challenges regarding precision oncology is drug coverage for non-FDA-approved actionable mutations, most of which are not covered by insurance providers. It will be challenging to obtain approval from these private insurance companies for such cases, whereas coverage will be easier to obtain (but not guaranteed) from public-funded insurance. In this regard, the molecular tumor board’s role is critical to making individualized recommendations for such cases. Currently, there is no molecular tumor board in the UAE. Most oncologists rely on their experience to make clinical decisions and their connections with other institutions with molecular tumor boards abroad to seek advice when facing such a clinical scenario. We recommend establishing a UAE-wide molecular tumor board to improve the precision of oncology practice and cancer patients’ outcomes in the UAE.

The only dedicated precision oncology clinic was established at Burjeel Medical City in May 2021.

The Emirati Genome Program, launched in July 2021, is an initiative focused on providing preventive and personalized healthcare for UAE citizens, aiming to sequence the genome of the UAE population. The general Emirati population was positive about biomedical research and optimistic about the potential value of the genome program and biobank [63].

A successful program outcome will equip healthcare practitioners with high-quality information that will enable them to provide advanced diagnosis, treatment options, and personalized and preventive programs tailored to an individual’s unique genetic makeup. It will also help predict and prevent present and future genetic diseases and implement new therapies for rare and chronic diseases, including cancer [64].

To advance precision oncology in the UAE, a collaborative effort is required from stakeholders and UAE-level policymaking, payers, healthcare providers, and patient advocacy groups. To make precision oncology affordable and improve cancer patient outcomes in the UAE, NGS testing and indications must be standardized, with interoperable infrastructure, funding, data, and research [65].

## 21. Pathology, Molecular, and Cytogenetics Testing

Before initiating cancer therapy, a diagnostic tumor tissue sample evaluated in a pathology laboratory by a pathologist must confirm the malignancy type and provide key prognostic factors that direct the treatment offered [66]. In the UAE, there are variations in pathology reporting quality, depending on the pathologist’s training and experience. Because most laboratories are accredited by the College of American Pathologists (CAP), tumor checklists are mandatory for reporting cancer histopathology. CAP tumor checklists ensure a minimum pathology dataset to be reported in a synoptic report. Accredited laboratories are also expected to have key quality measures in place for physician competency and to expect the benchmarking of performance in report categories such as cytology, cervical smears, and thyroid FNAs. CCCs tend to have better and more reliable pathology reporting than smaller clinics and hospitals. Performance is improved with the presence of multidisciplinary tumor (MDT) boards. There are examples of international collaboration with published audited data on pathology discrepancies and the revision of primary diagnoses.

There is expertise in the UAE, with various subspecialties in sociopathology with one to two larger groups. Overall, these organ-specific expert practices are performed at different hospitals and laboratories and are not centralized. The number of organ specialists boarded in their subspecialty would be limited to the American Boards (hematopathology, cytopathology, dermatopathology, neuropathology) and the Royal College UK. One of the most pressing unmet needs is cytogenetic and molecular diagnostic testing, which is not easily accessible or available in the UAE, and many specialized tests are sent abroad to international centers in the USA and Europe, resulting in significant delays in diagnosis and treatment initiation (2–4 weeks) in many cases. There is a need for centralized pathology reporting for complex suspected cancer cases, and the utilization of digital pathology can facilitate second opinions from specialized cancer centers abroad. Mandating a second pathology reading to confirm malignancies is expected in accredited labs. The second review of pathology in cancer treatment programs contributes to the quality of treatment. Even though significant changes are rare (usually 3%), minor changes can be significant [67,68]. Soft tissue and brain tumors increasingly require molecular data, and these should have integrated reports with appropriate information and tumor classification. In our opinion, recent advances in HER2 testing and their impact on therapy should require a mandatory second review, as inconsistencies can lead to errors in clinical management [69,70].

A review of USA malpractice data recognized melanoma, breast, and thyroid fine needle aspiration (FNA) as the major pathology reports with the highest risk for a lawsuit. An audit of national data can also prioritize high-risk areas for improvement and mitigation via a 2nd and/or expert review [69,70,71].

Our recommendations for improving pathology, molecular, and cytogenetic testing are as follows:

1. With the increasing adoption of digital pathology, UAE pathologists can access a larger pool of experts with an improved turn-around time for reports. A second pathologist should review all first diagnoses of malignancies. The preference is for one with organ-specific expertise.
2. Trained experts should review high-risk and specific organ pathologies. Centers of excellence for specific organ types should be created or identified to ensure an adequate volume of cases is seen by organ-specific specialists. These should include reviews of melanomas by dermatopathologists, brain tumors by neuropathologists, lymphoma/leukemias by hematopathologists, and thyroid FNAs by cytopathologists [62,64,65]. Likewise, limited numbers of central and reference laboratories should offer treatment-related testing such as HER2 FISH and NGS.
3. The Dubai Health Authority (DHA) and/or the Department of Health Abu Dhabi should audit standard quality metrics and pathologist competencies.
4. The central pathology review process should be considered as we develop more research protocols.
5. The UAE should encourage the establishment of biobanking facilities to facilitate future research efforts.

## 22. Availability and Cost of Cancer Drugs

Cancer drugs, including the latest approved medications, are widely available in the UAE. The UAE approval process has been very swift, and many drugs are being approved soon after being FDA-approved. For instance, in the case of a relatively new drug for lung cancer, sotorasib, the UAE was the second country worldwide to approve the drug after the USA [72]. The issue arises when the mother company does not register some drugs, and in this case, the procurement process becomes lengthy (4–6 weeks) and expensive.

Oncology biosimilars have shown comparable efficacy and safety based on clinical evidence and physicochemical quality data in real-world settings [73]. With the increasing cost of cancer drugs worldwide, biosimilars have been widely utilized to somewhat control the increasing cost of cancer drugs. Many biosimilars are approved in the UAE; their utilization varies between physicians and cancer centers. We recommend the usage of approved biosimilar cancer drugs as part of the cancer center formulary.

## 23. Oncology Nursing

The oncology nurse shortage is not new, and COVID-19 has exacerbated the shortage of oncology nurses [74]. The UAE is no different from the rest of the world when it comes to the issue of a shortage of oncology nurses. Most oncology nurses in the UAE are from Jordan, the Philippines, India, and Lebanon. The nurses tend to circulate in the cancer centers in the UAE with better pay. More recently, the UAE has dropped the two years of experience required to become licensed in the UAE in a move to attract more nurses to join the nursing workforce in the UAE.

In the United Arab Emirates, the Oncology Nursing Society (EOHNS) is the official body representing the UAE’s oncology nurses, with its main activities including CME participation. The EOHNS’s mission is to provide a community for cancer nurses in the UAE, with the aim of improving nursing care for cancer patients and their families, promoting the role of nurses in cancer care, and developing nursing leaders to shape the future of oncology nursing care. There are no structured training programs for oncology nursing training in the UAE. We recommend establishing a training program for oncology nursing to face the shortage of nurses in the UAE.

Furthermore, the role of a nurse practitioner is not very well established in the UAE. There may be a challenge in implementing such a position due to the current medical practice laws, as well as challenges with patients and their families in the UAE as they are used to being taken care of by a physician rather than a nurse; furthermore, there is an excess of oncologists in the UAE, and thus the need for nurse practitioners may not be as well established as it is in the USA, which has a shortage of oncologists.

The EOHNS has been leading oncology nurses to develop oncology nursing research and evidence-based practice, which is reflected in its mission: “to promote excellence in oncology nursing research and quality cancer care to cancer patients in the UAE.” The EOHNS has advocated for oncology nursing research and specified two tracks in their annual conference for nursing research and evidence-based practice. Developing an advanced practice role in oncology nursing in the UAE will foster and enhance oncology nursing research and evidence-based practice. The SEHA identifies and addresses research issues, attracts high-quality research nurses, supports research activities, and promotes the visibility of nursing research in Abu Dhabi. The nursing research committee has adopted a nursing research development program across all SEHA facilities to facilitate the education of registered nurses regarding evidence-based practice and research processes, emphasizing oncology nursing research studies.

We recommend that oncology nurses be encouraged to engage in cancer research with research courses and incentives for employment promotions across the UAE.

## 24. Artificial Intelligence and Cancer Care in the UAE

The UAE adopted artificial intelligence (AI) in cancer care earlier than other countries in the region. The IBM Watson oncology program was piloted in Tawam hospital in 2016 and was planned to aid clinical decision-making in clinics’ day-to-day cancer management. The project was then terminated when IBM suspended the Watson program as the AI technology used in Watson did not live up to expectations.

Currently, multiple AI platforms are being used in the UAE, mainly for aiding cancer diagnosis in imaging, e.g., breast and lung cancer screening. Table 9 summarizes the current AI approaches used in clinical practice in the UAE.

The UAE has also been contributing to AI and cancer research. A team of researchers from New York University (NYU) in the USA and NYU Abu Dhabi has reportedly developed a new AI system to identify breast cancer in ultrasound images. The system offers “radiologist-level accuracy” and has been designed as a decision-support tool for clinicians. The findings, published in *Nature*, showed that the system would help radiologists significantly decrease their “false positive” and requested biopsy rates while maintaining the same level of sensitivity [75,76]. A group of researchers from Mohamed bin Zayed University for AI has also published their work on developing machine learning algorithms that can predict cancer type classifications from multi-omics data [77].

## 25. COVID-19 and Cancer Care in UAE

The UAE has been a role model in managing the COVID-19 pandemic with low COVID-19-related mortality, with the first worldwide phase 3 COVID-19 vaccination clinical trial; a 100% vaccination rate for the eligible population; the continuation of all medical services for acute and chronic medical conditions during the pandemic; and finally, by providing medical aid, ventilators, testing kits, personal protective equipment, and supplies to 135 countries worldwide. The UAE has also been voted one of the best countries to live in during the pandemic [78].

Early in the pandemic, many cancer patients returned to the UAE from abroad, and all were accommodated to resume treatment. In many countries, sixty-eight cancer services had been interrupted. However, cancer care has continued in the UAE; for example, chemotherapy infusion units, radiation therapy sessions, cancer surgeries, and oncology clinic visits have all continued uninterrupted throughout the entire UAE [79]. This has been largely the effect of strong communication by the Emirates Oncology Society, which encoured oncologists and healthcare providers to continue providing cancer care despite the pandemic through educational webinars and campaigns [80].

There has been a significant contribution to cancer care and COVID-19 research from the UAE. The first international recommendations for cancer care during the pandemic were published early during the pandemic and have received more than 650 citations since then and were awarded the publication of the year (2020) by an oncologist journal in the USA [81,82]. It has also been listed as one of the most cited articles from the Arab world during the pandemic [83]. Other notable publications include the first pre-chemotherapy COVID-19 screening study, which was published in *JAMA Oncology* and was one of the trending articles at the time of its publication [84,85]. Other publications from the UAE have also been published in regional and international journals [86,87,88,89,90,91].

## 26. Research

**Figure 2:**
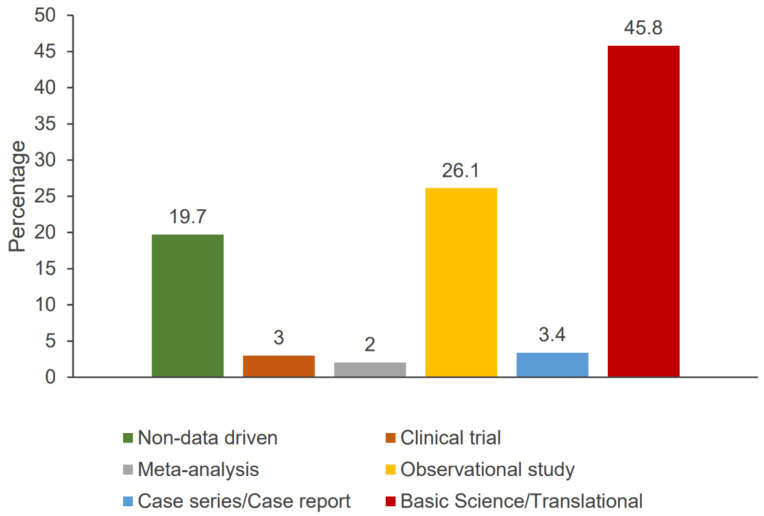
Cancer research output in the UAE by the type of the publication over the 20-year period from 2001 to 2021.

We performed a dedicated and systematic literature search to identify publications related to breast cancer from the UAE. We searched PubMed using the terms “breast” AND “Cancer* OR Oncol* OR malignant* OR tumor OR tumor” AND “emirates OR UAE” on 8 August 2021. A total of 203 journal publications by authors from the UAE were retrieved, with the earliest publication being from 2001. The majority were basic science/translational (45.8%) or observational (26.1%) studies, whereas 40 (19.1%) were non-data-driven publications (e.g., reviews, consensus statements, and editorials). Only six clinical trials were identified (Figure 2). Of note, among the 163 data-driven publications, only 62 (38%) were performed in the UAE.

In contrast, the remaining studies were performed abroad, with most authors having dual affiliations in the UAE and centers outside the UAE, reflecting a high rate of international collaboration [92].

In general, efficiency in cancer research is developing, though numerous evidence gaps remain. With the continuous growth of academic institutions and research programs committed to cellular and molecular research initiatives, the scope for basic as well as translational research is typically improving in the UAE. The primary focus of observational studies has been on basic epidemiological and screening parameters. Future perspectives should emphasize the expansion of national registries, along with the longitudinal assembly of clinically pertinent variables, which can update the molecular and clinical profile of breast cancer in a better way in the UAE and, more crucially, provide information on survival metrics that can support management strategies. Finally, there is an apparent deficiency of therapeutic clinical trials, an issue mirrored by the rest of the region.

There have been few previous attempts to initiate large, randomized trials. However, these have been primarily hampered by challenges with accruals, indicating a need for better public and provider awareness about the role of clinical trials in offering access to innovative medicines in cancer.

Since the resource and regulatory infrastructures are progressive and adaptable to international requirements and standards, various attempts are underway to strengthen collaborations with clinical trial sponsors to organize interventional studies in the UAE [93].

**Figure 3:**
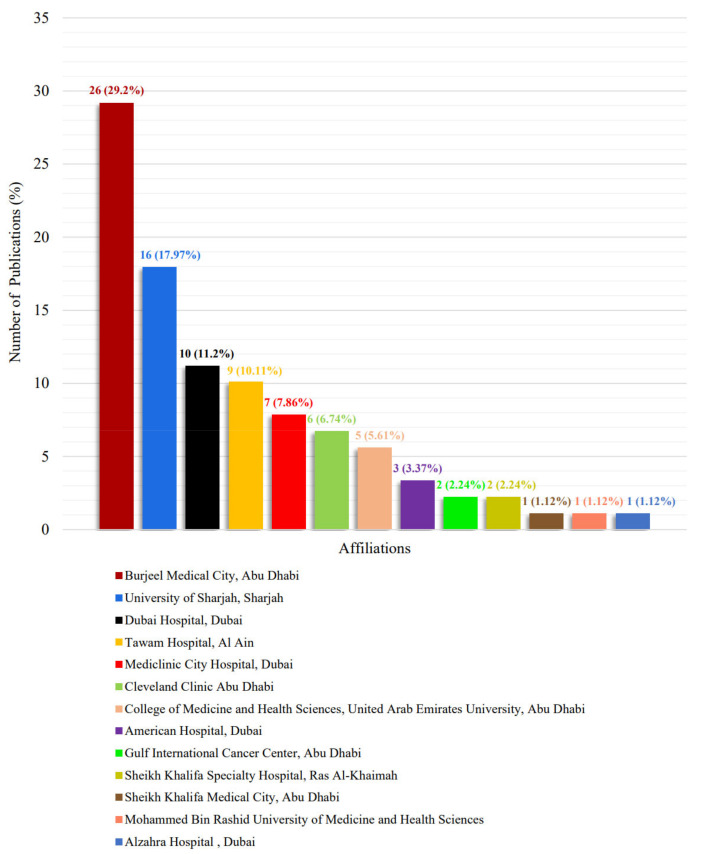
The ranking of cancer centers based on their cancer research output in 2021.

The ranking of cancer centers based on their cancer research output in 2021 is outlined in Figure 3 [94].

## 27. Education and Training

Well-structured advanced fellowship training in oncology and hematology is lacking in the UAE and is an area of unmet need. The only accredited program by the Accreditation Council for Graduate Medical Education–International (ACGME-I) in the UAE is the medical oncology fellowship training program that started in August 2019. Three fellows were enrolled then, and as of yet, no program graduates have been announced. The program is a three-year medical oncology fellowship (not including hematology). The hematology fellowship training program was started in Dubai in 2020 and is the only hematology fellowship training program in the UAE.

The National Institute for Health Specialties (NIHS) was established by Cabinet Decree No. 28 of 2014 as a national institution, mandated to spearhead, regulate, and organize professional development for the health workforce with a particular emphasis on specialty training. The NIHS is nested within the United Arab Emirates University, the country’s premier national higher education institution, and is governed by a Board of Directors, chaired by the Ministry of Education, UAE. The NIHS oncology committee is chaired by Professor Humaid Alshamsi and has 12 members. The committee was established in May 2022 and aimed to formulate the NIHS Program Requirements for Specialty Education in Hematology and Medical Oncology (Emirati Board in Hematology and Medical Oncology) for the graduates of the programs to hold tier-one qualifications as consultants in the UAE.

Currently, there is no oncology residency training for nurses in the UAE. There is an urgent need to establish well-structured oncology training programs for physicians and nurses, especially considering the global shortage of trained nurses in oncology [74].

## 28. Emirates Oncology Society

The Emirates Oncology Society (EOS) is the official organization representing oncology healthcare providers in the UAE [95]. It operates under the Emirates Medical Association (EMA) umbrella. The EMA was established in 1981 as a non-profit organization composed of health practitioners that are members of the EMA, as defined by its laws. The EMA supervises and conducts scientific training, events, and conferences and collaborates with health organizations. Its headquarters are in Dubai. EMA operates under the Ministry of Social Affairs.

The EOS was established in 2016 and was not functional until 2020. It has more than 80 medical, surgical, and radiation oncology members. A member must have an active UAE license in the field of oncology.

The EOS has published more than 50 peer-reviewed articles over the last three years, becoming the most active scientific society in the UAE. The society has also conducted more than 200 CME hours with activities directed at healthcare providers and the public. The EOS annual conference is held annually in September of each year. The EOS annual award is the first of its kind for individuals and organizations with a positive impact globally, regionally, and nationally, including the EOS lifetime achievement awards.

The EOS also plays an essential part as an advisor to regulators. For example, members of the EOS have been advising the Dubai Health Authorities and the Ministry of Health and Prevention, UAE, regarding the best practices for better-quality cancer care and as members of the national committee for cancer control.

## 29. Cancer Continuing Medical Education (CME) in the UAE

Different organizations and the pharmaceutical industry run many oncology educational activities across the UAE and throughout the year. The International Oncology Conference started in 2011 by VPS Healthcare, and The International Oncology Conference by Tawam Hospital started in 2011 as well. The Excellence in Oncology Care conference in Dubai started in 2010, and the Emirates Oncology Society annual conference started in 2020. The International Oncology Summit, organized by the American hospital in Dubai, started in 2019. In addition, pharmaceutical industry CMEs are delivered with participation from oncologists from top universities and institutions around the globe.

## 30. Medical Tourism for Cancer Care in the UAE

The UAE is a desirable destination for healthcare, especially for African and Iraqi patients. The UAE’s health system is ranked 27th in the world by the WHO [86]. The UAE government has launched medical tourism portals that allow international patients to book procedures and access a wide range of tourism services, such as direct contact with healthcare providers, visa issuance, booking appointments, hotels, transportation, and other recreational activities [87].

One of the key aspects that makes the UAE a hub for medical tourism is the central location of the UAE; its international centers and hospitals; the healthcare expertise, with US and European training and experience, among doctors practicing in the UAE; and the rapid availability of new cancer drugs that are not available in many countries, making the UAE an attractive choice for many cancer patients. One of the barriers is cost, with treatment tending to be more expensive than in other countries that also provide cancer medical tourism, e.g., India. More efforts are needed to make cancer medical tourism more affordable to attract more cancer patients, especially for niche indications such as bone marrow transplantations.

## 31. Government-Funded Cancer Care Medical Tourism outside the UAE

The UAE spent an estimated over USD 163 million in 2013 on government-funded cancer care, medical treatment abroad, and medical tourism outside the UAE [96]. There are no published official data available about the stages or types of cancer cases treated abroad. The five top destinations for cancer care medical tourism among people from the UAE are the United States of America, the Federal Republic of Germany, the Republic of Singapore, the Republic of Korea (South Korea), and the Kingdom of Thailand [97,98]. One study in which administrative data were obtained from the DHA for UAE nationals who sought medical treatment overseas during 2009–2016 included data from 6557 UAE nationals. The top three treatment destinations were Germany (46%), the UK (19%), and Thailand (14%). The most common medical specialties were orthopedic surgery (13%), oncology (13%), and neurosurgery (10%). Adjusted for a number of covariates, oncology exhibited the highest expected number of trips (IRR 1.34, 95% CI: 1.24–1.44) [99].

There are multiple independent sponsoring agencies for cancer care abroad in the UAE, including all the health authorities (DOH, DHA, and Ministry of Health and Prevention), Presidential Affairs offices, armed forces, police, charity organizations, and self-paying [2]. The sponsoring requirements and processes vary between the different sponsoring agencies, with essential requirements including being a UAE citizen. However, exceptions are also given for non-UAE citizens in some cases if they can document that the required treatment is unavailable in the UAE. Nevertheless, despite the availability of cancer treatments in the UAE, many patients receive exemptions from local treatment to be treated abroad. There are no uniform guidelines or criteria for these entities/agencies to guide patient selection for treatment abroad [2].

In an internal review of 273 patients who requested to travel abroad between January and September 2017 at a tertiary referral oncology center in the UAE, 86% of the referrals were not clinically indicated based on the availability of these oncology services in the UAE [2]. This was before the availability of bone marrow transplantation services, as many of these cases were BMT cases [6]. Based on our experience, we estimate that more than 95% of cancer cases are eligible to be treated in the UAE.

As we outlined above in the section regarding medical tourists coming to the UAE for cancer care, the UAE has very well-established cancer care centers, yet there are factors which lead patients to seek treatment abroad [98]:

- The patient may deny the diagnosis and look for a second opinion to confirm the diagnosis;
- Patients and their families may believe that there are newer treatment options for cancer abroad (drugs not available in the UAE, new technologies, and expertise only available abroad); and
- The patient and their families may receive a generous government allowance while away, in addition to the patient’s full paid leave while on sick leave abroad; this sick leave is not as flexible for patients treated locally in the UAE. The same applies to family members who also receive companion leave while abroad, but this is not the case if the patient is treated locally.

Traveling abroad for cancer care is not sustainable in the long term. To make recommendations to help reduce the demand and need for cancer care abroad, focused research is required to identify the motivations for traveling abroad and barriers to receiving treatment locally. As discussed in our previous report [91], treatment abroad should be limited to complex cancer cases requiring specialized care abroad that is unavailable in the UAE after a consensus review by the accredited comprehensive cancer centers. Promoting public trust in cancer care within the UAE is an important aspect that needs special attention and outreach programs at the national level, with engagement from regulators and sponsoring agencies for traveling abroad.

## 32. National Cancer Control Plan

Currently, no national cancer control plan has been implemented in the UAE. We have previously published a proposed national cancer control plan, highlighting the plan’s key elements [93]. A comprehensive and effective cancer control plan requires accurate data; a reliable cancer registry; and periodic monitoring, assessment, and evaluation. The UAE’s cancer control plan was aligned with the WHO and EMRO frameworks, with defined goals and objectives. The main objectives are to combat cancer, reduce the incidence, improve outcomes and quality of life for cancer patients, and reduce mortality. There is also a focus on improving prevention, early detection, prompt diagnosis, treatment facilitation, public health education, continuity of care, performance evaluation, training of the workforce, and cancer research.

## 33. Integrative Oncology

Integrative oncology is defined as a patient-centered, evidence-informed field of cancer care that utilizes mind and body practices, natural products, and/or lifestyle modifications from different traditions alongside conventional cancer treatments [100]. Integrative oncology is a new specialty in the UAE, with no cancer center providing this unique service within their centers. Despite being controversial, some cancer patients from the UAE travel abroad specifically for integrative cancer treatment. There are multiple homeopathic centers that treat cancer patients with complementary medicine in the UAE. The specialty needs to be regulated and monitored to avoid misuse in patients with cancer. The integrative oncology specialty will be an important field to be added to the cancer care landscape in the UAE.

## 34. Major Recommendations for the Advancement of Cancer Care in the UAE

**Figure 4:**
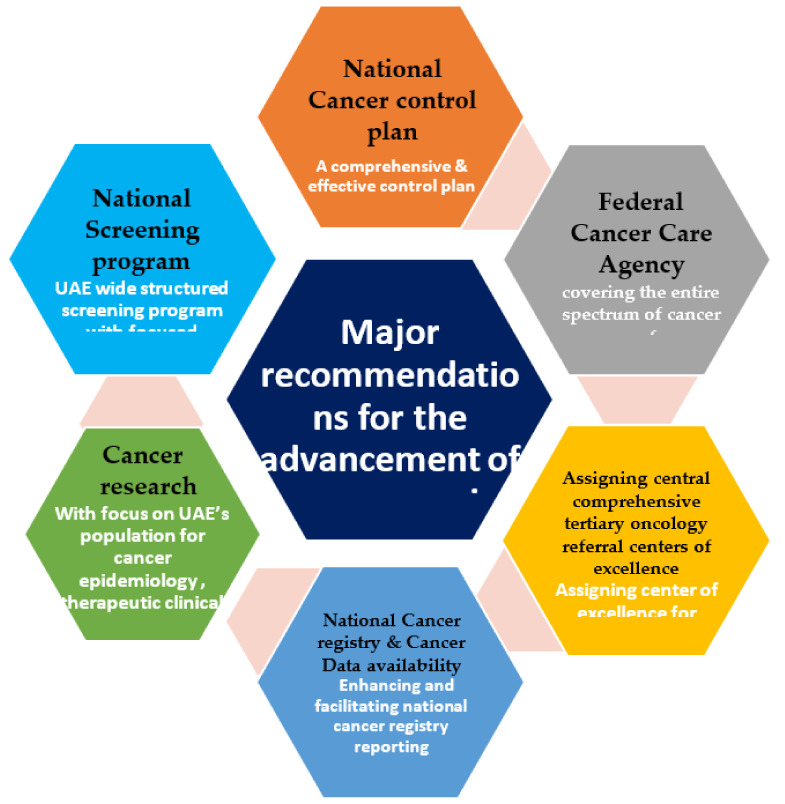
Major recommendations for the advancement of cancer care in the UAE.

To advance cancer care in the UAE to the next level of the best care, we have outlined the top major recommendations in Figure 4.

This includes the following recommendations:

- A national cancer control plan (CCP): a comprehensive and effective control plan requires accurate data, a reliable cancer registry, and periodic monitoring and evaluation.
- A Federal Cancer Care Agency covering the entire spectrum of cancer care, from prevention and screening to diagnosis, establishing treatment guidelines, and ensuring that cancer patients across the UAE receive safe, evidence-based, effective, and high-quality treatment.
- Assigning central comprehensive tertiary oncology referral centers of excellence.
- Assigning centers of excellence for centers meeting a strict criteria to improve the outcomes of cancer patients in the UAE.
- Making national cancer registry and cancer data available by enhancing and facilitating national cancer registry reporting.
- Cancer research with a focus on UAE’s population for cancer epidemiology, therapeutic clinical trials, and international research collaboration.
- A national screening program for the UAE—a wide-structured screening program with focused research on barriers for screening, increasing public awareness.

## 35. Conclusions

In this review, we attempted to address the current status of cancer care in the UAE. We identified a swift revolution in cancer care in the UAE with the development of state-of-the-art cancer centers, likely more cancer centers than are needed for a small country. The next phase of cancer care should focus on quality control measures implemented by regulators across the UAE. A federal cancer agency is recommended, as cancer care requires a specialized governor. A national UAE cancer control program is much needed in order to improve early detection, screening, and appropriate referrals to cancer networks. Education and training are required to face the global shortage of oncology healthcare providers, especially oncology nurses. The activation of survivorship programs focusing on onco-fertility, psycho-oncology, precision oncology, and UAE cancer-specific research with regional and international collaboration is also essential for the next phase of cancer care in the UAE.

## Figures and Tables

**Table 1:** Top ten most common malignant primary sites among the UAE population, 2019.

Primary Site	Number of Malignant Cases 2019	%
Breast	883	20.2%
Thyroid	501	11.4%
Colorectal	413	9.4%
Skin	278	6.3%
Leukemia	220	5.0%
Non-Hodgkin lymphoma	215	4.9%
Prostate	172	3.9%
Bronchus and Lung	151	3.4%
Lip, Oral cavity & pharynx	142	3.2%
Uterus	125	2.9%

Source: Ministry of Health and Prevention, Statistics and Research Center, National Disease Registry—UAE National Cancer Registry Report 2019 [1].

**Table 2:** Top ten most common malignant primary sites among females and males, 2019.

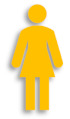		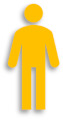	
Primary Site	%	Primary Site	%
Breast	36.1%	Colorectal	13.1%
Thyroid	15.5%	Skin	9.3%
Colorectal	6.5%	Prostate	8.8%
Uterus	5.2%	Leukemia	7.1%
Ovary	4.1%	Non-Hodgkin lymphoma	6.7%
Skin	4.0%	Thyroid	6.4%
Cervix Uteri	3.7%	Bronchus and Lung	5.3%
Non-Hodgkin lymphoma	3.5%	Lip, Oral cavity & pharynx	5.3%
Leukemia	3.4%	Urinary Bladder	4.8%
Bronchus and Lung	1.9%	Kidney & Renal pelvis	4.2%

Source: Ministry of Health and Prevention, Statistics and Research Center, National Disease Registry—UAE National Cancer Registry Report 2019 [1].

**Table 3:** Distribution of malignant cancer deaths by type of cancer in UAE, 2019.

Primary Site	%
Malignant Neoplasm of Breast	11.5%
Malignant Neoplasm of Colon	8.9%
Malignant Neoplasm of Trachea, bronchus and Lung	8.8%
Leukemia	4.4%
Malignant Neoplasm of Stomach	4.7%
Malignant Neoplasm of Cervix Uteri	0.6%
Malignant Neoplasm of Rectum	1.1%
Other Malignant Neoplasm	59.9%
Grand Total	100.0%

Source: Ministry of Health and Prevention, Statistics and Research Center, National Disease Registry—UAE National Cancer Registry Report 2019 [1].

**Table 4:** Cancer screening programs in the UAE.

Program	Geographical Coverage	Early Detection Cancer Covered	Implementation Date	Outcome Data	Outcome Data	Current Status
Simply Check	Emirate of Abu Dhabi	Breast, Colorectal, Cervical Cancers and lung cancer	Sep 2014	Citizens only	Not published	Merged into IFHAS program
ITMENAN *	Northern Emirates	Breast, Colorectal and Cervical Cancers	Nov 2016	Citizens only	Not published	Active–ongoing
WEQAYA *	Emirate of Abu Dhabi	Breast, Colorectal and Cervical Cancers	Jan 2021	Citizens only	Not published	Merged into IFHAS program
IFHAS *	Emirate of Abu Dhabi	Breast, Colorectal, Cervical Cancers and lung cancer	June 2022	Citizens only	Ongoing-Not published	Active–ongoing
Dubai Health Authority cancer screening program	Emirate of Dubai	Breast, Colorectal and Cervical Cancers	Nov 2017	Citizens only	Ongoing-Not published	Active–ongoing
Cancer Patient Support Program (BASMAH *)	Emirate of Dubai	Breast, Colorectal and Cervical Cancers	Nov 2020	Non-citizens only	Ongoing-Not published	Active–ongoing

* ITMENAN means contentment in Arabic, Weqaya means prevention in Arabic, IFHAS means CHECK in Arabic, BASMA means Smile in Arabic.

**Table 5:** Established comprehensive cancer centers in the UAE.

Hospital	Location	Established	Oncology International Accreditation	Unique Services	Services Not Offered
American Hospital Dubai	Dubai	2010	JCI Clinical Care Program Certification in 2017 National Accreditation Program for Breast Centers (NAPBC) in 2015	Only acute hematology in private sector and BMT unit in Dubai Palliative care unit Pediatric Oncology	Research unit/publication, Genetic Counseling
Burjeel Medical City	Abu Dhabi	2020	The European Society for Medical Oncology (ESMO) Designated Centres of Oncology and Palliative Care, the only centre accredited by ESMO in the UAE	BMT unit & Only pediatric BMT in UAE Only palliative care service in Abu Dhabi city Only acute hematology in private sector in Abu Dhabi Cancer research unit Only Brain lab in UAE Pediatric Oncology	Genetic Counseling
Mediclinic City Hospital	Dubai	2016	JCI accredited breast cancer unit	Brachytherapy Palliative care unit Pediatric Oncology	Acute hematology and BMT Unit, Genetic Counseling
Tawam Hospital ^ψ^	Alain	1979	JCI accredited breast cancer unit	Palliative service Genetic Counseling Pediatric Oncology	Hepatobiliary surgery BMT Unit Research & publications

The following services must be available at the facility to be considered as a comprehensive cancer centers: medical adult and pediatric oncology and hematology; surgical oncology; radiation oncology; nuclear medicine and palliative care. ^ψ^ The Positron emission tomography (PET) scanner which is located near the main building of Tawam hospital is affiliated with Cleveland Clinic Abu Dhabi’s (CCAD).

**Table 6:** Distribution of the top five pediatric cancer cases by primary sites in UAE, 2019.

Primary Site	Number of Pediatric Cancer Cases	%
Leukemia	44	35.2%
Brain & CNS	14	11.2%
Connective and Soft Tissue	9	7.2%
Non-Hodgkin Lymphoma	9	7.2%
Bone and Articular Cartilage	7	5.6%

Source: Ministry of Health and Prevention, Statistics and Research Center, National Disease Registry—UAE National Cancer Registry Report 2019 [1].

**Table 7:** Hospitals providing paediatric oncology in the UAE.

Hospital	City	
American Hospital Dubai	Dubai	Private Hospital
Burjeel Medical City	Abu Dhabi	Private Hospital
Dubai Hospital	Dubai	Public Hospital
NMC Hospital Abu Dhabi	Abu Dhabi	Private Hospital
Mediclinic City Hospital	Dubai	Private Hospital
Sheikh Khalifa Medical City	Abu Dhabi	Public Hospital
Tawam Hospital	Al Ain	Public Hospital

**Table 8:** Cancer support programs and cancer support groups in the UAE.

	Funded By	Location	Sevice Provided
Friends of Cancer Patients (FoCP)	Charity	Sharjah	Financial support
Cancer Patient Support Program (BASMAH)-ISAHD	Dubai government	Dubai	Oncology Medication support
Cancer Patient Care Society RAHMA	Charity	Abu Dhabi	Financial support
Emirates Cancer Society (Previously known as Moazzara)	Charity	Al Ain	Financial support
UAE Access Programs—Axios International	Medications support	Dubai	Oncology Medication support
Various Charity Organizations in UAE	Treatment $ coverage ($ allowed per case varies)	Across the UAE	Financial support
Brest Friends	N/A	Dubai	Psychological support
Majlis Al Amal—Al Jalila Foundation	N/A	Dubai	Psychological support For female cancer patients
The Cancer Majlis	N/A	Dubai	Psychological support
Bosom Buddies	N/A	Abu Dhabi	Psychological support

**Table 9:** Current status of clinical and research initiatives in the UAE.

AI Technology	Facility	Year	Format	Status
IBM™ Watson Oncology—Pilot	SEHA—Tawam Hopital	2016	Clinical decision in oncology	Suspended
AI enabled Digital mammography system, Lunit INSIGHT MMG Lung Cancer Screening—Coreline—Medical AI solutions	International Radiology Centre—Sharjah Commercial	2021	AI-enabled independent reader for breast cancer screening and lung cancer screening	Active
Prognica Labs	Dubai Commercial	2021	Prognica Labs Uses Artificial Intelligence To Detect Masses In Mammography Screenings	Active
Mammography Intelligent Assessment (Mia)™	UAE Commercial	2021	first and only AI-enabled independent reader for breast cancer screening to be commercially available in the UAE	Active
The GI Genius™ intelligent endoscopy module	Sheikh Shakhbout Medical City Abu Dhabi	2021	is the first-to-market, computer-aided polyp detection system powered by AI	Active
Khalifa University researchers	Research Abu Dhabi	2021	To identify cancer in tissue samples, which could speed up diagnosis and improve outcomes in patients with colorectal cancer.	Active
DoH—Abu Dhabi	Research Abu Dhabi	2022	First Personalised Precision Medicine for oncology in collaboration with Mubadala Health, Cleveland Clinic Abu Dhabi, NYU Abu Dhabi, Mohamed bin Zayed University of Artificial Intelligence and G42 Healthcare.	Active
Mohamed bin Zayed University of Artificial Intelligence team	Research Abu Dhabi	2022	AI tool to better diagnosis and treatment of pancreatic cancer	Active

## Data Availability

Not applicable.

## Appendix A

**Table A1:** Number of cancer cases among the UAE population according to primary site, gender, and nationality in 2019.

Primary Site	UAE Citizens			Non-UAE Citizens			Total
	Female	Male	Total	Female	Male	Total	
All invasive cancers (Malignant Cases)	674	443	1117	1751	1513	3264	4381
Lip, Oral cavity, and pharynx	11	20	31	28	83	111	142
Esophagus	2	3	5	5	18	23	28
Stomach	9	20	29	15	45	60	89
Small Intestine	2	3	5	8	11	19	24
Colorectal	63	66	129	94	190	284	413
Liver and intrahepatic bile ducts	9	17	26	10	36	46	72
Gallbladder, other and unspecified part of biliary tract	5	5	10	8	13	21	31
Pancreas	4	10	14	13	28	41	55
Nasal cavity, middle ear, accessory sinuses		1	1	4	4	8	9
Larynx		11	11		20	20	31
Bronchus and Lung	10	31	41	37	73	110	151
Bone and articular cartilage	2	5	7	9	13	22	29
Skin melanoma	1	2	3	22	26	48	51
Skin	7	12	19	90	169	259	278
Mesothelioma	1		1	3	4	7	8
Kaposi sarcoma					2	2	2
Retroperitoneum and peritoneum	1		1	8	2	10	11
Connective and soft tissue	5	3	8	15	15	30	38
Breast	209	1	210	666	7	673	883
Cervix uteri	22		22	68		68	90
Uterus	46		46	79		79	125
Ovary	24		24	76		76	100
Prostate		57	57		115	115	172
Testis		8	8		31	31	39
Kidney and Renal pelvis	9	20	29	24	63	87	116
Ureter and other urinary organs		3	3		2	2	5
Urinary bladder	7	20	27	10	73	83	110
Eye	1	2	3	1	4	5	8
Brain and CNS	6	10	16	28	48	76	92
Thyroid	119	26	145	256	100	356	501
Other endocrine glands	2		2	2	8	10	12
Unknown or unspecified sites	9	4	13	15	29	44	57
Hodgkin’s lymphoma	15	10	25	25	26	51	76
Non-Hodgkin’s lymphoma	32	32	64	52	99	151	215
Multiple myeloma	5	5	10	10	22	32	42
Leukemia	27	25	52	55	113	168	220
Other hematopoietic malignancies	3	3	6	6	9	15	21
Other malignancies	6	8	14	9	12	21	35
Non-invasive cancers (In situ cases)	54	22	76	125	51	176	252
Carcinoma in situ of oral cavity, oesophagus and stomach		1	1		4	4	5
Carcinoma in situ of other and unspecified digestive organs	3	1	4	2	2	4	8
Carcinoma in situ of middle ear and respiratory system		2	2	1	3	4	6
Melanoma in situ		1	1	8	12	20	21
Carcinoma in situ of skin		2	2	5	7	12	14
Carcinoma in situ of breast	24	1	25	68	2	70	95
Carcinoma in situ of cervix uteri	24		24	36		36	60
Carcinoma in situ of other and unspecified genital organs				1	4	5	5
Carcinoma in situ of other and unspecified sites	3	14	17	4	17	21	38
Grand Total	728	465	1193	1876	1564	3440	4633

Source: Ministry of Health and Prevention, Statistics and Research Center, National Disease Registry—UAE National Cancer Registry Report 2019 (https://mohap.gov.ae/assets/download/f3eb10c9/CANCER%20INCIDENCE%20IN%20UNITED%20ARAB%20EMIRATES%20ANNUAL%20REPORT%20OF%20THE%20UAE%20-%202019.pdf.aspx, accessed on 10 August 2022).

## Appendix B

**Table A2:** Cancer centers and hospitals in the UAE in alphabetical order.

Cancer Center	Location	Facility Available								
		Medical Oncology and Infusion Unit	Acute Hematology Service	Bone Marrow transplantation Unit	Pediatric Oncology	Radiation	Surgical Oncology	Nuclear Medicine/PET Imaging	Palliative Care Unit	Research Unit
Abu Dhabi Stem Cells Center	Abu Dhabi	No	No	**Yes**	No	No	No	No	No	No
Advanced Care Oncology Center	Dubai	**Yes**	No	No	No	No	No	No	No	No
American Hospital Dubai	Dubai	**Yes**	**Yes**	**Yes**	**Yes**	**Yes**	**Yes**	**Yes**	**Yes**	No
Al Zahra Hospital Dubai	Dubai	**Yes**	No	No	No	No	**Yes**	No	No	No
Aster Hospital	Dubai	**Yes**	No	No	No	No	**Yes**	No	No	No
Burjeel Hospital	Abu Dhabi	**Yes**	No	No	No	No	**Yes**	No	No	No
Burjeel Hospital for Advanced Surgery Dubai	Dubai	**Yes**	No	No	No	No	**Yes**	No	No	No
Burjeel Medical City	Abu Dhabi	**Yes**	**Yes**	**Yes**	**Yes**	**Yes**	**Yes**	**Yes**	**Yes**	**Yes**
Burjeel Day Surgery Center, Al Reem Island	Abu Dhabi	**Yes**	No	No	No	No	No	No	No	No
Burjeel Royal Hospital	Alain	**Yes**	No	No	No	No	**Yes**	No	No	No
Burjeel Specialty Hospital	Sharjah	**Yes**	No	No	No	No	**Yes**	No	No	No
Canadian Hospital	Dubai	**Yes**	No	No	No	No	**Yes**	No	No	No
Cleveland Clinic Abu Dhabi	Abu Dhabi	**Yes**	No	No	No	No	**Yes**	**Yes ** * **(Offsite)**	No	No
Clemenceau Medical Center	Dubai	**Yes**	No	No	**Yes**	No	**Yes**	**Yes**	No	**Yes**
Dubai Hospital	Dubai	**Yes**	**Yes**	No	**Yes**	No	**Yes**	**Yes**	No	**Yes**
Gulf International Cancer Center	Abu Dhabi	**Yes**	No	No	No	**Yes**	No	**Yes**	No	No
Lifeline Hospital VPS	Abu Dhabi	**Yes**	No	No	No	No	No	No	No	No
King’s College Hospital London, Dubai	Dubai	**Yes**	**Yes**	No	No	No	**Yes**	No	No	No
Medcare hospital	Sharjah	**Yes**	No	No	No	No	**Yes**	No	No	No
Mediclinic Airport Road	Abu Dhabi	**Yes**	**Yes**	No	No	**Yes**	**Yes**	No	No	No
Mediclinic City Hospital	Dubai	**Yes**	**Yes**	No	**Yes**	**Yes**	**Yes**	**Yes**	**Yes**	**Yes**
Neuro Spinal Hospital	Dubai	**Yes**	No	No	No	**Yes**	**Yes**	No	No	No
NMC Royal Hospital Sharjah	Sharjah	**Yes**	No	No	No	No	**Yes**	No	No	No
NMC Specialty Hospital	Abu Dhabi	**Yes**	No	No	Yes	No	**Yes**	No	No	No
Saudi German Hospital Dubai	Dubai	**Yes**	No	No	No	**Yes**	**Yes**	No	No	No
Saudi German Hospital Ajman	Ajman	**Yes**	No	No	No	No	**Yes**	No	No	No
Sharjah University Hospital	Sharjah	**Yes**	No	No	No	No	**Yes**	No	No	**Yes**
Sheikh Khalifa Specialty Hospital	Ras al-Khaimah	**Yes**	No	No	No	**Yes**	**Yes**	**Yes**	No	No
Sheikh Shakhbout Medical City	Abu Dhabi	**Yes**	**Yes**	No	No	No	**Yes**	No	No	No
Tawam Hospital	Alain	**Yes**	**Yes**	No	**Yes**	**Yes**	**Yes**	No *	**Yes**	**Yes**
Yas Clinic	Abu Dhabi	**Yes**	No	No	No	No	No	No	No	No
Zulekha Hospital Sharjah	Sharjah	**Yes**	No	No	No	No	**Yes**	No	No	No

* Cleveland Clinic Abu Dhabi’s (CCAD) positron emission tomography (PET) scanner is located in Al Ain city (150 km away from the CCAD main campus) near the main building of Tawam hospital.
